# Binding of hnRNP I–vRNA Regulates Sindbis Virus Structural Protein Expression to Promote Particle Infectivity

**DOI:** 10.3390/v14071423

**Published:** 2022-06-28

**Authors:** Claire E. Westcott, Shefah Qazi, Anna M. Maiocco, Suchetana Mukhopadhyay, Kevin J. Sokoloski

**Affiliations:** 1 Department of Microbiology and Immunology, School of Medicine, University of Louisville, Louisville, KY 40202, USA; claire.jones@louisville.edu; 2 Department of Biology, Indiana University—Bloomington, Bloomington, IN 47405, USA; shefahq@gmail.com (S.Q.); sumukhop@indiana.edu (S.M.); 3 Center for Predictive Medicine and Emerging Infectious Diseases, School of Medicine, University of Louisville, Louisville, KY 40202, USA; ammaiocco@indiana.edu

**Keywords:** Sindbis virus, hnRNP I, PTBP1, RNA-binding, protein tethering

## Abstract

Alphaviruses cause significant outbreaks of febrile illness and debilitating multi-joint arthritis for prolonged periods after initial infection. We have previously reported that several host hnRNP proteins bind to the Sindbis virus (SINV) RNAs, and disrupting the sites of these RNA–protein interactions results in decreased viral titers in tissue culture models of infection. Intriguingly, the primary molecular defect associated with the disruption of the hnRNP interactions is enhanced viral structural protein expression; however, the precise underlying mechanisms spurring the enhanced gene expression remain unknown. Moreover, our previous efforts were unable to functionally dissect whether the observed phenotypes were due to the loss of hnRNP binding or the incorporation of polymorphisms into the primary nucleotide sequence of SINV. To determine if the loss of hnRNP binding was the primary cause of attenuation or if the disruption of the RNA sequence itself was responsible for the observed phenotypes, we utilized an innovative protein tethering approach to restore the binding of the hnRNP proteins in the absence of the native interaction site. Specifically, we reconstituted the hnRNP I interaction by incorporating the 20nt bovine immunodeficiency virus transactivation RNA response (BIV-TAR) at the site of the native hnRNP I interaction sequence, which will bind with high specificity to proteins tagged with a TAT peptide. The reestablishment of the hnRNP I–vRNA interaction via the BIV-TAR/TAT tethering approach restored the phenotype back to wild-type levels. This included an apparent decrease in structural protein expression in the absence of the native primary nucleotide sequences corresponding to the hnRNP I interaction site. Collectively, the characterization of the hnRNP I interaction site elucidated the role of hnRNPs during viral infection.

## 1. Introduction

Alphaviruses are positive-sense, single-stranded RNA viruses that have, and will likely continue to, cause significant outbreaks of clinically severe disease [1,2,3]. A primary reason for the sustained emergence of mosquito-borne viruses may largely be due to the wide geographical distribution of competent mosquito vectors aggravated by climatological change and global trade, which have led to the dissemination of vector mosquitos [4,5,6]. Based on clinical presentation, there are two subgroups of the alphaviruses, namely the encephalitic and arthritogenic subgroups. Sindbis virus (SINV), Chikungunya virus (CHIKV), Ross River virus (RRV), Semliki Forest virus (SFV), and Mayaro virus (MAYV) are all considered to be members of the arthritogenic group, and infection can result in moderate to severe febrile illness often followed by long-term multi-joint arthritis, which may persist for several years past the resolution of acute infection [7,8,9]. While the arthritogenic alphaviruses are not typically as deadly as those of the encephalitic subgroup, the arthritogenic alphaviruses still cause significant burdens to community health systems and reduced quality of life to infected individuals [10,11]. As stated earlier, due to the widespread distribution of vector-competent mosquitos, the majority of the world’s population is at risk for at least one alphaviral infection. Despite there being significant clinical disease, there are no FDA-approved treatments or safe, effective vaccines to limit the public health burden of the alphaviruses.

The identification and study of host protein interactions with viral RNAs or viral proteins is not a novel concept, and many studies have identified host factors with known RNA-binding properties [12,13,14,15,16,17,18,19,20,21,22,23,24,25,26,27,28]. While these prior efforts have established the importance of these factors to alphaviral biology, many have overlooked the potential impact of the host RNA-binding proteins engaging with the viral RNAs (vRNAs) on viral biology and have instead utilized RNAi or gene knockout studies to evaluate the importance of specific host factors to infection. In addition to not directly defining the importance of the protein–vRNA interaction, this approach has the disadvantage of potentially disrupting the host system if the target protein is deeply involved in the regulation of host RNA biology [29,30,31,32,33]. Thus, it remains possible that the knockdown or knockout of host factors essential to cellular homeostasis may cause artefacts to viral replication. 

Previously, we published a study that determined that there are several host hnRNP proteins that directly bind to the SINV vRNAs in a site-specific manner [34]. It was found that disrupting the hnRNP binding sites in the alphaviral RNAs led to decreased growth kinetics, and surprisingly this phenotype correlated largely with increased structural protein expression. Nonetheless, whether the phenotypes observed following the mutation of the hnRNP interaction sites was specifically due to the loss of hnRNP–vRNA binding or due to the mutations in the primary nucleotide sequences or RNA secondary structures remained unknown. The primary goal of this study was to determine whether the observed phenotypes were genuinely ascribable to the loss of hnRNP protein binding through the reconstitution of the protein–RNA interaction in the absence of the native interaction site. To this end, we employed a modified protein tethering approach to develop a mutant SINV, where a native hnRNP interaction site was replaced with the bovine immunodeficiency transactivating response RNA element (TAR) [35,36,37]. As the inclusion of the BIV-TAR element would alter the primary amino acid sequence of the target, we prioritized the hnRNP I interaction for evaluation, as the hnRNP I interaction site is located in the SINV 3′UTR [34]. Importantly, the inclusion of the BIV-TAR element enabled the direct assessment of the importance of the hnRNP–vRNA binding to SINV infection. Altogether, our data indicate that the loss of hnRNP I protein binding to the vRNA is directly responsible for the phenotype observed following the mutation of the native interaction site. Furthermore, the data from these efforts further define the biological and molecular importance of the hnRNP proteins to alphaviral infection.

## 2. Materials and Methods

### 2.1. Tissue Culture Cells

BHK-21 (ATCC CCL-10) and HEK293 (ATCC CRL-1573) tissue culture cells were cultured in minimal essential medium (MEM; Cellgro Mediatech, Inc., Manassas, VA, USA) supplemented with 10% fetal bovine serum (FBS; Corning, Corning, NY, USA), 1× penicillin–streptomycin (Pen/Strep; Corning, Corning, NY, USA), 1× nonessential amino acids (NEAA; Corning, Corning, NY, USA), and l-glutamine (Corning, Corning, NY, USA). HEK293T cells were cultured in Dulbecco’s modified Eagle’s medium (Corning, Corning, NY, USA) supplemented with 10% fetal bovine serum, 1× penicillin–streptomycin, 1× nonessential amino acids, and 5 mM L-glutamine. All cells were maintained at 37 °C in a humidified incubator at 5% CO_2_.

Where specifically noted, tissue culture dishes receiving HEK293 cells were pre-treated with poly-l Lysine (Advanced Biomatrix, Carlsbad, CA, USA) to aid cell adherence and prevent premature detachment during handling. Briefly, tissue culture dishes were pre-treated with 0.1 mg/mL poly-l Lysine for 30 min at 4 °C. After poly-l lysine treatment, the stock solution was removed and the wells were briefly rinsed twice with 1× PBS and allowed to dry under sterile conditions prior to seeding the dishes with HEK293 cells for use the next day.

### 2.2. Sindbis Virus Mutant Construction and Preparation

To generate a SINV mutant with the native hnRNP I interaction site replaced with the 21nt BIV-TAR element, we utilized a two-step mutational approach. First, using site-directed mutagenesis, the primary nucleotide sequence of the native hnRNP I interaction site of SINV.TE12-nanoluciferase, consisting of nucleotides 11,557 to 11,586, was replaced with a *NotI* restriction digestion site to generate the hnRNP I interaction-deficient SINV.hnRNP I^Δ^ [38]. After sequencing to confirm the veracity of the clone, a restriction enzyme/DNA ligase strategy was utilized to insert the BIV-TAR element into the *NotI* (New England Biolabs, Ipswich, MA, USA) site of the hnRNP I interaction-deficient subclone. The specific sequence of the BIV-TAR element, including the *NotI* restriction enzyme sequences and flanking sequences, was 5′-gcggccgcaacactGGCTCGTGTAGCTCATTAGCTCCGAGCCtatcctgcggccgc-3′, with the BIV-TAR-specific sequences capitalized for reference. The resulting virus, SINV.hnRNP I^TAR^, was sequenced to confirm the presence of the BIV-TAR element and to verify that the orientation of the element was correct. 

All viruses utilized in this study were generated via the electroporation of in-vitro-transcribed RNAs derived from cDNA infectious clones, as previously described [39]. Briefly, approximately 10 ug of in-vitro-transcribed RNA was electroporated into BHK-21 cells by a single pulse from a Gene Pulser Xcell electroporation system set to deliver a single square-wave discharge of 125 V for a period of 12.50 ms. After the development of significant cytopathic effects, the tissue culture supernatants were harvested and clarified of cell debris via centrifugation prior to aliquoting and storage at −80 °C for later use. 

### 2.3. Control and hnRNP I^TAT^ Transfection of HEK293 Cells

To reconstitute the hnRNP I interaction via the BIV-TAR/TAT system and test the importance of the hnRNP I interaction to SINV infection, HEK293 cells were transfected with an expression plasmid encoding the full-length hnRNP I protein with a c-terminal TAT peptide tag (pEXPR.hnRNPI-TAT) using Lipofectamine 3000 (Invitrogen, Thermo Fisher Scientific, Waltham, MA, USA), according to the manufacturer’s instructions. Transfections were conducted in a 12-well format at 80% confluence. Each well was transfected with DNA–lipid complexes generated by mixing 0.5 ug DNA supplemented with 2 uLof P3000 reagent and 1.5 uL of Lipofectamine 3000 reagent in separate volumes of 50 uL of Optimem (Thermo Fisher Scientific, Waltham, MA, USA). Control transfections lacking the hnRNP I expression clone were conducted in parallel. The cells were transfected in a minimal volume of 1 ml of whole growth medium and allowed to incubate overnight prior to replacing the media with fresh growth medium before continuing with further experimentation. 

### 2.4. Quantitative Immunoprecipitation of hnRNP I–vRNA Complexes

Transfected HEK293 cells were infected with either wild-type SINV or SINV.hnRNP I^TAR^ at a multiplicity of infection (MOI) of 5 plaque-forming units (PFU) per cell in a 12-well format. At 16 h post-infection (hpi), the tissue culture monolayers were harvested via gentle scraping and centrifugation at 300× *g* for five minutes. The media were aspirated and the cells were washed with 1× phosphate-buffered saline (PBS; Corning, Corning, NY USA) to remove contaminating media. The washed cell pellets were gently resuspended in 1×PBS supplemented with 1.0% formaldehyde and incubated under gentle agitation for 7 min. The cross-linked cell pellets were then recollected via centrifugation at 1000× *g* for 3 min, and the supernatant was promptly removed and replaced with 1×PBS supplemented with 0.25 M glycine to quench any excess formaldehyde. After a 5 min incubation, the cells were again collected via centrifugation as above and resuspended in 400 ul of RIPA buffer (50 mM Tris pH 7.6, 150 mM NaCl, 1.0% NP-40, 0.5% sodium deoxycholate, 0.1% SDS) and lysed via brief sonication, as previously described [34,40].

The resulting lysates were clarified via high-speed centrifugation (5 min at 16,000× *g*) to remove insoluble debris, and subsequently immunoprecipitated with 10 uL of either anti-hnRNP I (anti-PTBP1; rabbit polyclonal; PA5-95949; Thermo Fisher Scientific, Waltham, MA, USA) or anti-NALP1 (rabbit polyclonal; PA5-20005; Thermo Fisher Scientific, Waltham, MA, USA) as a nonspecific control. Antibody complexes were then precipitated from the lysate via the addition of paramagnetic protein G agarose beads. The beads were washed a minimum of five times prior to the elution of the immunoprecipitated materials via incubation at 70 °C for 30 min. The total RNA was extracted from the eluate using TRIzol reagent (Promega, Madison, WI, USA), as indicated by the manufacturer’s instructions. 

The purified RNAs were used as the inputs for the synthesis of cDNA for analysis by qRT-PCR, as previously described. The relative quantitative immunoprecipitation was determined by comparing the amount of viral RNAs detected across the indicated experimental conditions, after normalization to the sample specific inputs and nonspecific control immunoprecipitations as determined by qRT-PCR. 

### 2.5. Analysis of Viral Growth Kinetics 

The viral replication kinetics were assayed using one-step growth kinetics assays in HEK293 cells bound to poly-l lysine plates. After transfection the cell, monolayers were infected with either wild-type SINV or SINV.hnRNP I^TAR^ at an MOI of 10 PFU per cell. After a one-hour adsorption period, the cells were carefully washed twice with 1×PBS prior to the addition of whole medium supplemented with 25 mM HEPES to enable the use of an automated liquid handling system lacking a CO_2_ atmosphere. At the indicated times post-infection, the cell supernatant was collected and stored at 4 °C, and fresh replacement media was added. Viral titers were determined via plaque assay using BHK-21 cells overlaid with a 2% Avicel (FMC, Philadelphia, PA, USA) suspension (in whole media). After a 30 h incubation period, the samples were fixed with 3.7% formaldehyde (in 1×PBS) and visualized by crystal violet staining. 

### 2.6. Quantitative Analysis of SINV Structural Protein Expression

The assessment of structural protein expression was performed as previously described, with several specific modifications [34]. Briefly, HEK293 cells were cultured on poly-l-lysine-treated plates and transfected as described above. The tissue culture monolayers were then infected with either wild-type SINV or SINV.hnRNP I^TAR^ at a MOI of 10 PFU per cell. After removal of the unbound virus particles, fresh tissue culture medium was added and the cells were incubated under normal conditions. At the indicated times post-infection, the supernatant was removed and discarded and the cell monolayers were washed with 1×PBS. Whole-cell lysates were then harvested by scraping in 1×PBS supplemented with 0.15% Triton X-100 (Avantor; Radnor Township, PA, USA). The lysates were collected in microcentrifuge tubes and frozen at −80 °C. After the completion of the time course, the cell lysates were thawed, vortexed, and clarified via centrifugation at 17,000× *g* for 3 min to remove insoluble materials. Equivalent amounts of cell lysate, as confirmed by Bradford assay (Avantor; Radnor Township, PA, USA), were then assessed using the Nano-Glo nanoluciferase assay system (Promega, Madison, WI, USA) according to the manufacturer’s instructions. The nanoluciferase activity was detected in a BioTek Synergy H1 microplate reader. 

### 2.7. Quantification of Viral RNA Synthesis or Accumulation and Particle Numbers

The lysates generated for the quantitative assessment of structural protein expression, as described above, were treated with TRIzol reagent and extracted using a Direct-zol-96 MagBead RNA kit (Zymol Research; R2102; Irvine, CA, USA) via a Kingfisher Duo Prime automated nucleic acid extractor system. The quantitative detection of the individual RNA species was accomplished using strand-specific reverse transcription and standard curve qRT-PCR, as previously described [34]. The RNA levels were normalized to the 18S rRNA levels. 

The particle numbers, as determined by genome equivalents per ml, were quantitatively assessed similarly to those described previously, and as generally described above, with two major differences. First, the input materials consisted of tissue culture supernatants that had been boiled prior to the synthesis of genome-specific cDNAs. Second, the samples were not normalized to an endogenous control transcript and were instead normalized through the use of equal volumes. 

### 2.8. Purification of SINV Particles, Morphological Assesments via Transmission Electron Microscopy, and SDS-PAGE 

The concentration and purification of SINV particles were adapted from the low-speed, low-temperature centrifugation protocol [41]. Briefly, HEK293 cells were cultured in 100 mm dishes infected (2 dishes per virus, per prep) to 95% confluence. The monolayers were then infected with either wild-type or SINV.hnRNP I^TAR^ at a MOI of 5 PFU units per cell. After the adsorption period, the inoculum was removed and replaced with Virus Production Serum-Free Media (VP-SFM; Gibco, Thermo Fisher Scientific, Waltham, MA, USA) supplemented with 1× penicillin–streptomycin, 1× nonessential amino acids, and 5 mM L-glutamine. After a 20 h incubation period, the supernatants were harvested and clarified via centrifuge to remove cell debris. The clarified supernatants were then transferred to Oakridge tubes and the virus particles were gently pelleted via centrifugation at 5300× *g* for 18 h at 4 °C. After centrifugation, the tubes were promptly removed, the supernatant was carefully decanted, and the residual moisture was gently blotted with a Kimwipe wrapped around a pipette tip. The pellets were resuspended in HEPES-NaCl-EDTA resuspension buffer (HNE; pH = 7.5; 20 nM HEPES, 150 mM NaCl, 0.1 mM EDTA). 

For the transmission electron microscopy (TEM) analysis, the SINV particles were applies to Formvar- and carbon-coated 400-mesh copper grids and stained with 1% uranyl acetate. The prepared grids were imaged using a JEOL 1010 transmission electron microscope operating at 80 kV. The images were recorded via a Gatan Ultrascan 4000 CCD camera. The image processing and the measurement of the particle diameter were performed in ImageJ.

The compositional assessment of SINV particles was accomplished via standard SDS-PAGE with nonspecific Coomassie staining. Equal particle numbers of either wild-type or SINV.hnRNP I^TAR^ were boiled in 2× Laemmli buffer prior to the resolution of proteins by molecular weight via SDS-PAGE on 10% pre-cast gels (Criterion^TM^ TGX^TM^; Bio-Rad Laboratories, Hercules, CA, USA). After electrophoresis, the gels were stained using Coomassie blue and visualized using a flatbed scanner. 

### 2.9. Quantitative Assessment of Viral Attachment

The HEK293 cells from untreated plates were scraped, aspirated, and transferred into sterile microfuge tubes. After ensuring that the cells were evenly resuspended, the cell aliquot was evenly divided and inoculated with either wild-type or SINV.hnRNP I^TAR^ at a MOI of 0.1 PFU per cell and incubated with gentle mixing at 4 °C to allow binding but not entry of the viral particles. After the incubation period, one aliquot was immediately treated with TRIzol to generate an input sample. The cells in the second aliquot were gently pelleted via centrifugation at 300× *g* for 5 min at 4 °C, and extensively washed three times with excess volumes of 1×PBS. Prior to being treated with TRIzol reagent, the cell pellets were resuspended in an equivalent volume (relative to the input control) of whole media. The total RNA from input and bound samples was extracted as described above, and the number of viral particles bound to the host cells was determined via qRT-PCR, as described above.

To determine the relative efficiency with which each viral particle population bound to the host cell, the percent binding was calculated for each specific pair by comparing the input and bound samples. A comparative analysis of binding was performed by normalizing the percent bound to that detected for wild-type particles. For simplicity, comparisons were restricted to host cell derivation (as per mock or hnRNP I^TAT^-transfected cells). 

### 2.10. Deglycosylation of Viral Particles

Viral particles were deglycosylated via treatment with PNGase F (Recombinant; New England Biolabs, Ipswich, MA, USA) under nondenaturing reaction conditions. Briefly, equal amounts of viral particles were diluted into PNGase nondenaturing reaction buffer, which was pre-prepared as close to 1× as possible to prevent the destruction of the viral particles due to osmotic pressure. The mixtures were then split into two parallel reactions, and 1% of total reaction volume of PNGase F was added to one reaction. Both samples were then incubated for a minimum of 18 h at room temperature prior to the determination of the viral titer via serial dilution assays. 

### 2.11. Statistical Analyses 

All quantitative data shown are from a minimum of three independent biological replicates, unless more replicates are specifically indicated. Data shown represent the quantitative mean, with the error bars representing the standard deviation of the means. Where appropriate, a statistical analysis of the ratios was performed using variable bootstrapping, as described previously [42]. Pairwise statistical analyses were conducted using unpaired Student’s t-tests, with a minimum threshold *p*-value of < 0.05 being considered statistically significant. A statistical analysis of the viral growth kinetics was accomplished using an area under the curve (AUC) analysis. 

## 3. Results

### 3.1. Developing a Protein Tethering System to Study the Impact of hnRNP I Binding to SINV RNAs

On the basis of our prior data, we concluded that the disruption of hnRNP–vRNA interaction sites, and ergo the loss of hnRNP–vRNA binding, resulted in decreased viral growth kinetics, potentially as the result of increased structural protein expression during SINV infection. However, from these data, conclusions could not be made as to whether this phenotype was due to the direct loss of hnRNP binding to the viral RNA, or due to some other consequence of mutating the primary nucleotide sequences of the interaction sites themselves. As such, we sought to develop a system by which the protein–RNA interaction of the hnRNP proteins could be functionally restored in the absence of the native interaction site to address whether hnRNP–vRNA binding or a cryptic feature of the nucleotide primary sequence or structure was primarily responsible for the observed defects in growth kinetics following the disruption of the hnRNP–vRNA interaction sites. 

In our previous study, we identified the hnRNP–vRNA interaction sites between hnRNP K, hnRNP I, and hnRNP M and the SINV viral RNAs using next-generation sequencing approaches [34]. The interaction sites for the hnRNP K and hnRNP M proteins were found within the structural ORF coding region of the viral subgenomic RNA, whereas the interaction site for hnRNP I was determined to be in the viral 3′UTR. Due to the constraints associated with manipulating the coding regions of the viral RNAs, we elected to continue these studies by focusing on the hnRNP I interaction site because of its location in the 3′UTR, as this region of the genome has a greater degree of sequence plasticity. 

**Figure 1:**
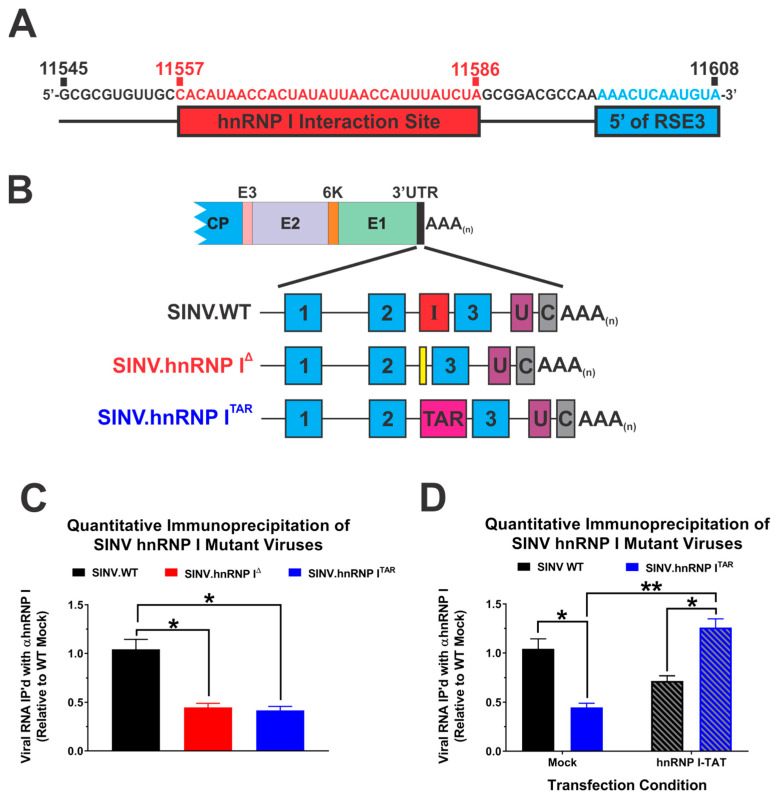
Protein tethering restores hnRNP I protein binding in the absence of the native interaction site. (**A**) A nucleotide map of the hnRNP I interaction site in the SINV TE12 3′UTR as defined by prior CLIP-Seq efforts. The specific sequences targeted for deletion in this study are highlighted in red, and sequences belonging to RSE3 (which were included in the original deletion mutant) are highlighted in cyan. (**B**) A schematic diagram of the viruses used in these studies, including wild-type SINV (SINV.WT) and the hnRNP I interaction-deficient mutants SINV.hnRNP I^Δ^ and SINV.hnRNP I^TAR^, which incorporated a bovine immunodeficiency virus transactivation response element (BIV-TAR) in lieu of the native interaction site. The SINV repeat sequence elements (RSEs) are denoted by cyan boxes with their relative number labeled inside, similarly the hnRNP I interaction site, the SINV U-rich element and 19-nt 3′ conserved sequence element are indicated with red, purple, and gray boxes labeled with an I, U, or C, respectively. Elements are drawn to scale. (**C**) Immunoprecipitation of vRNA–hnRNP I complexes derived from mock-transfected HEK293 cells infected with the indicated viruses. (**D**) Immunoprecipitation of vRNA–hnRNP I complexes derived from hnRNP I^TAT^-transfected HEK293 cells infected with the indicated viruses. Quantitative detection of vRNA relative to the SINV.WT level was accomplished using qRT-PCR. Quantitative data shown are the means of three independent infections or co-immunoprecipitations, with the error bars representing the standard deviation of the means. Statistical significance, as determined by Student’s *t*-test, is indicated above the specific comparisons (with * ≤ 0.05; ** ≤ 0.01).

Our previous approach to eliminate the hnRNP I interaction relied on the deletion of the entire interaction site as identified by way of CLIP-Seq. Specifically, in the original hnRNP I interaction mutant, nucleotides 11,545 to 11,608 were deleted from the SINV 3′UTR. While the majority of this nucleotide range exists between the repeat sequence elements (RSEs) 2 and 3, the tail end of the original hnRNP I interaction deletion mutant included approximately 12 nt of RSE3. Thus, as detailed above, the phenotype observed with the original hnRNP I interaction site mutant could be due to either the loss of hnRNP I binding, the disruption of sequences or structures important to the alphaviral biology, or a combination of the two possibilities. As the primary goal of this study was to functionally dissect the importance of hnRNP I binding from the viral RNA sequence, we developed a new set of mutants to determine the specific impacts of the hnRNP I–vRNA interaction. These mutants utilized a more focused definition of the hnRNP I interaction site, as depicted in Figure 1A, to avoid altering the sequence and putative structures of the RSEs. 

To determine the specific impact of hnRNP I binding on SINV infection, we employed a modified protein tethering approach that binds the hnRNP protein to the vRNA in a targeted manner in the absence of the native interaction site or sequence. As diagrammed in Figure 1B, the native SINV hnRNP I interaction site was replaced with the 20 nucleotide bovine immunodeficiency virus transactivation response element (BIV-TAR) sequence to create SINV.hnRNP I^TAR^. Importantly, in addition to ablating the native hnRNP I interaction site, the TAR element enables the site-specific tethering of proteins tagged with a bovine immunodeficiency transactivator (TAT) peptide motif [35]. Thus, by expressing an hnRNP I protein tagged with the TAT peptide motif (hnRNP I^TAT^) we may reconstitute the hnRNP–vRNA interaction, enabling direct comparisons of infections with the native hnRNP I interaction, no hnNRP I interaction, and a forced hnRNP I interaction to determine the explicit importance of hnRNP–vRNA binding.

First, to confirm that the BIV-TAR/TAT system reestablished the interaction between the viral RNA and hnRNP I, we quantitatively assessed the interaction via immunoprecipitation. To this end, cells were either mock-transfected or transfected with an expression plasmid encoding the hnRNP I^TAT^ fusion protein, and then infected with either wild-type SINV, SINV.hnRNP I^Δ^, or SINV.hnRNP I^TAR^. At 16 h post-infection, the cells were crosslinked with formaldehyde and whole-cell lysates were generated via the addition of detergent and gentle sonication [43]. RNA–protein complexes were immunoprecipitated via an hnRNP I-specific antibody, and the amount of viral RNA that co-immunoprecipitated with hnRNP I was determined via qRT-PCR. To ensure the specificity, the quantitative detection of the vRNAs was normalized to parallel control immunoprecipitations using a nonspecific antibody. As shown in Figure 1C, the deletion of the previously identified hnRNP I interaction site (as per SINV.hnRNP I^Δ^ and SINV.hnRNP I^TAR^) negatively impacted the immunoprecipitation of SINV vRNA with anti-hnRNP I antibody by approximately 2-fold in comparison with the wild-type SINV (SINV.WT). In contrast, quantitative immunoprecipitations of hnRNP I protein–RNA complexes in lysates generated from HEK293 cells that were transiently transfected with an expression plasmid encoding the hnRNP I^TAT^ fusion protein indicated that the BIV TAR/TAT system was capable of reconstituting the hnRNP–vRNA interaction in the absence of the native interaction site. Specifically, as shown in Figure 1D, the co-immunoprecipitation of the SINV vRNA with hnRNP I antibody was significantly increased for SINV.hnRNP I^TAR^ in the presence of hnRNP I^TAT^ relative to SINV.WT and SINV.hnRNP I^TAR^ in the absence of hnRNP I^TAT^. Interestingly, the co-immunoprecipitation of SINV.WT vRNAs was modestly decreased in the presence of hnRNP I^TAT^. The precise underlying the reasons behind this phenomenon are unclear, but the potential causes of this decrease are speculated on in the Section 4 Discussion. 

Altogether, these data confirm that the BIV-TAR/TAT system is a means by which the interaction between the SINV vRNAs and the hnRNP I protein may be restored in the absence of the native interaction site. Nonetheless, while confirming that we may functionally dissect the binding from the vRNA primary sequence, the specific consequences of restoring the hnRNP I–vRNA interaction on the viral biology remain unaddressed.

### 3.2. Reconstitution of hnRNP I Binding Restores Growth Kinetics in Tissue Culture Models of Infection

**Figure 2:**
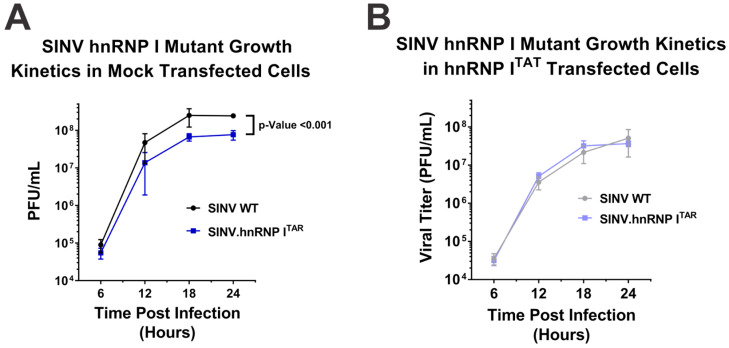
Restoration of hnRNP I binding results in wild-type-like growth kinetics. The capacity of the hnRNP–vRNA interaction site mutant viruses to replicate in HEK293 cells was assessed using one-step growth curves in (**A**) mock-transfected or (**B**) hnRNP I^TAT^-transfected cells. The titer was quantified using standard plaque assays. Quantitative data shown are the means of at least three minimum biological replicates, with the error bars representing the standard deviation of the means. Statistical significance, as determined by the area under the curve analysis, is shown above.

As the data above confirmed that the hnRNP I protein–RNA interaction could be reconstituted in the absence of the native interaction site via the BIV-TAR/TAT system, we next sought to examine whether hnRNP–vRNA binding impacted the viral growth kinetics. Briefly, HEK293 cells were either mock-transfected or transfected with an expression plasmid encoding hnRNP I^TAT^, and then subsequently infected with either SINV.WT or SINV.hnRNP I^TAR^ at a multiplicity of infection of 10 PFU/cell. Over a period of 24 h, the supernatants were collected every six hours and the viral titer was quantitatively determined using plaque assays. As shown in Figure 2A, the hnRNP I-binding-deficient mutant SINV.hnRNP I^TAR^ exhibited a statistically significant ~3.5-fold decrease in viral titer relative to the wild-type SINV. In contrast, when the hnRNP I interaction was restored through the BIV-TAR/TAT system in hnRNP I^TAT^-transfected cells, the viral growth kinetics observed for SINV.WT and SINV.hnRNP I^TAR^ were comparable (Figure 2B).

Interestingly, despite using parallel conditions for both the control transfection and hnRNP I^TAT^-transfected cells, the overall titers were lower for both viruses in the hnRNP I^TAT^-expressing cells. The precise underlying cause of this phenomenon is unclear; however, the overexpression of hnRNP I appears to negatively impact cellular homeostasis, as observed via the cell division and morphology (as shown in Supplementary Figure S1). 

**Figure 3:**
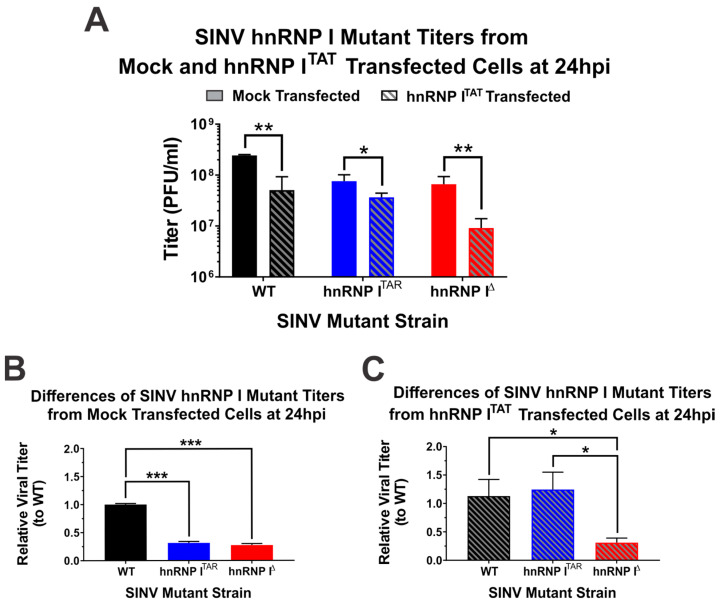
Specific reconstitution of hnRNP I–vRNA binding restores wild-type growth kinetics in a mutant lacking the native interaction site. (**A**) Viral titers of wild-type and hnRNP–vRNA interaction site mutant viruses SINV.hnRNP I^TAR^ and SINV.hnRNP I^Δ^ at 24 h post-infection of mock and hnRNP I^TAT^-transfected HEK293 cells infected at an MOI of 10 PFU per cell. (**B**) Comparative analysis of the viral titer for each of the aforementioned SINVs in mock-transfected HEK293 cells relative to wild-type SINV. (**C**) Identical to (**B**), with the exception that the HEK293 cells were transfected with hnRNP I^TAT^. Quantitative data shown are the means of at least three minimum biological replicates, with the error bars representing the standard deviation of the means. Statistical significance, as determined by Student’s *t*-test, are indicated above the specific comparisons (with * ≤ 0.05; ** ≤ 0.01; *** ≤ 0.001).

An unfortunate consequence of the apparent toxicity of hnRNP I^TAT^ overexpression is that critical assessments of the one-step growth kinetics data presented in Figure 2 do not enable the direct conclusion that reconstituting the hnRNP I interaction restores the wild-type-like growth kinetics. Indeed, an alternative conclusion could be that hnRNP I^TAT^ overexpression negatively impacted wild-type replication, while the replication of SINV.hnRNP I^TAR^ was unperturbed. To directly test whether SINV.hnRNP I^TAR^ improved to wild-type levels or wild-type deteriorated to meet SINV.hnRNP I^TAR^ levels, we assessed the impact of hnRNP I^TAT^ expression on the parental hnRNP I interaction site mutant SINV.hnRNP I^Δ^. As shown in Figure 3A, hnRNP I^TAT^ expression uniformly negatively impacted viral replication for all SINV mutants utilized in this study. Importantly, while growth differences were readily observed between wild-type SINV and both hnRNP I interaction-deficient viruses under mock-transfected conditions (Figure 3B), in the presence of hnRNP I^TAT^, both wild-type SINV and SINV.hnRNP I^TAR^ replicated to similar extents, while SINV.hnRNP I^Δ^ remained phenotypically distinct from wild-type SINV (Figure 3C). Thus, from these data, we are able to conclude that reconstituting the hnRNP I–vRNA interaction genuinely restored SINV.hnRNP I^TAR^ replication to wild-type levels. 

From these data and the previous section, we are able to conclude that replacing the native hnRNP I interaction site with the BIV-TAR element negatively impacts the viral growth kinetics in highly permissive tissue culture models of infection. More importantly, the reconstitution of the hnRNP–vRNA interaction via the BIV-TAR/TAT system in the presence of hnRNP I^TAT^ restored the wild-type-like growth kinetics, ultimately providing strong evidence that the direct loss of the interaction between the viral RNA and the hnRNP I protein is primarily responsible for the previously established phenotype. 

### 3.3. Binding of hnRNP I Correlates with Translational Repression of the SINV Subgenomic RNA

**Figure 4:**
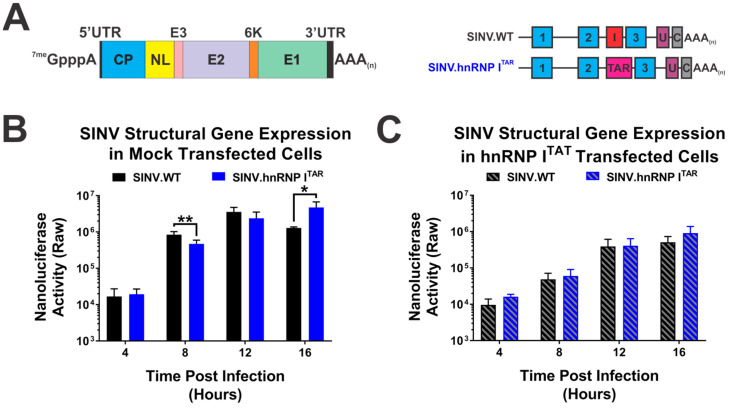
Restoration of hnRNP I binding abrogates the enhanced structural protein expression observed late during infection. (**A**) A graphic schematic of the nanoluciferase-based reporter strain derived from SINV TE12 that expresses nanoluciferase in parallel with the SINV Capsid protein during the translation of the subgenomic strand. (**B**) Mock-transfected or (**C**) hnRNP I^TAT^-transfected HEK293 cells were infected with the designated virus and nanoluciferase activity was quantified at the times indicated post-infection. Quantitative data shown are the means of three independent infections, with the error bars representing the standard deviation of the means. Statistical significance as determined by Student’s *t*-test is indicated above the specific comparisons (with * ≤ 0.05; ** ≤ 0.01).

As demonstrated by the data presented in Figure 1 and Figure 2, the BIV-TAR/TAT system is a means by which the specific impacts of hnRNP I binding to the viral RNAs may be assessed. Previously, we showed that disrupting the hnRNP I–vRNA interaction site resulted in increased structural protein expression; however, it was unknown whether the altered structural protein expression was specifically due to the loss of hnRNP I binding or the mutation of a cryptic regulatory element in the 3′UTR [34]. To delineate the impact of the hnRNP I binding on the structural protein expression, we utilized a reporter strain of SINV that expresses nanoluciferase from the subgenomic RNA strand (Figure 4A). Similar to what was previously reported, the loss of hnRNP I binding correlated with a biologically and statistically significant enhancement of SINV structural protein expression (Figure 4B). Indeed, at 16 hours post-infection (hpi), the subgenomic gene expression during the SINV.hnRNP I^TAR^ infection of HEK293 cells was significantly enhanced by approximately 4-fold relative to the wild-type SINV. However, at early times during the infection, this effect was notably absent, as at 4 hpi there was no difference in structural protein expression between SINV.WT and SINV.hnRNP I^TAR^. At both 8 and 12 hpi, the wild-type SINV exhibited slightly increased protein expression relative to SINV.hnRNP I^TAR^, yet only the difference observed at 8 hpi was found to be statistically significant. 

The examination of the structural protein expression during SINV infection after the reconstitution of the hnRNP I interaction via the BIV-TAR/TAT system revealed that the loss of hnRNP I binding was directly responsible for the enhancement of the structural protein expression late during infection. Specifically, in cells expressing hnRNP I^TAT^ there was no significant biological or statistical difference between wild-type or SINV.hnRNP I^TAR^ structural protein expression at any time (Figure 4C). Nonetheless, as observed during the analysis of viral growth kinetics above, the expression of hnRNP I^TAT^ reduced structural protein expression for both wild-type SINV and SINV.hnRNP I^TAR^_._

Together these data suggest that hnRNP I-binding is tied to the regulation of viral structural protein expression during infection, and that the enhancement of the structural protein expression due to the loss of hnRNP I binding is time-dependent and specific to the very late stages of infection.

### 3.4. Binding of hnRNP I Does Not Contribute to the Regulation of Viral RNA Synthesis

An established role for the hnRNP proteins during alphaviral infection centers around viral RNA synthesis; however, it should be noted that these studies relied upon RNAi-mediated knockdown strategies, which as described earlier could lead to substantial off-target impacts on the cellular environment [12,27,44]. Accordingly, to refine the understanding of the role of the hnRNP I protein in viral transcription and replication, we examined the RNA synthesis profiles of wild-type SINV and the hnRNP I interaction-deficient viruses during infections of HEK293 cells either mock-transfected or transfected with an hnRNP I^TAT^ expression plasmid. The detection of the individual viral RNA species was accomplished using standard qRT-PCR detection using previously reported methods over four-hour intervals from 4 hpi to 16 hpi. 

**Figure 5:**
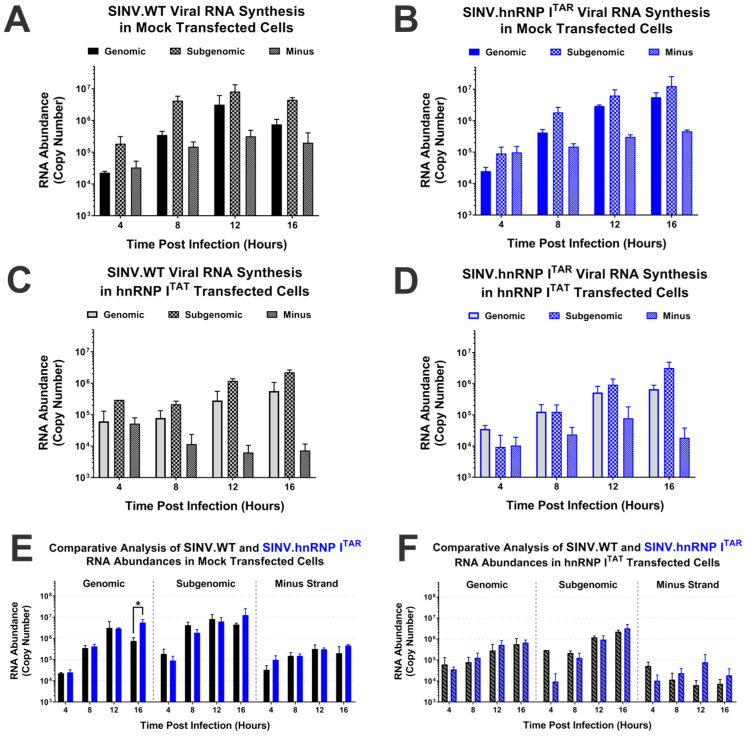
Viral RNA synthesis is not impacted by the hnRNP–vRNA interaction. Strand-specific quantitative analysis of the three SINV vRNA species in mock-transfected HEK293 cells infected with (**A**) SINV.WT or (**B**) SINV.hnRNP I^TAR^ viruses at an MOI of 10 PFU per cell. (**C**,**D**) Identical to the previously described panels, with the primary difference being that hnRNP I^TAT^-transfected HEK293 cells were used. (**E**,**F**) Data from the previous panels reconfigured to allow direct comparisons between the viruses in either cell condition. Quantitative data shown are the means of three independent infections, with the error bars representing the standard deviation of the means. Statistical significance as determined by Student’s *t*-test is indicated above the specific comparisons (with * ≤ 0.05).

As previously reported, the loss of the hnRNP I interaction did not significantly alter the synthesis or accumulation of the individual viral RNAs, as exhibited by the general RNA profiles observed for SINV.WT and SINV.hnRNP I^TAR^ with respect to time (Figure 5A,B) [34]. In contrast, as shown in Figure 5C,D, both SINV.WT and SINV.hnRNP I^TAR^ exhibited altered accumulation profiles in the presence of hnRNP I^TAT^. In the presence of hnRNP I^TAT^ expression, the synthesis and accumulation of both the genomic and subgenomic RNA species was negatively impacted, with average reduction rates of approximately 4- and 6-fold, respectively, for SINV.WT and SINV.hnRNP I^TAR^. Nonetheless, despite the clear impact of the hnRNP I^TAT^ expression on RNA synthesis, the overall magnitudes of the impact were similar. 

To enable a more direct comparison of the viral RNA species during SINV.WT and SINV.hnRNP I^TAR^ analyses, we assessed the quantitative data for the individual viruses using pairwise statistical analyses (Figure 5E,F). These analyses revealed that only a single pairwise sample was statistically different between SINV.WT and SINV.hnRNP I^TAR^, specifically the quantity of genomic viral RNA at 16 hpi in the mock-transfected condition. All other comparisons, including those for the subgenomic RNAs, were not different to any statistically significant degree (with a minimum α ≤ 0.05 on a one-tailed analysis). 

In summary, these data indicate that the synthesis and accumulation of viral RNA species is not negatively impacted by the loss of hnRNP I binding or the mutation of the native interaction sequence. However, the overexpression of hnRNP I negatively impacted the viral RNA synthesis and accumulation in a generalized manner. 

### 3.5. Binding of hnRNP I Is Important to the Viral Particle Function or Specific Infectivity

**Figure 6:**
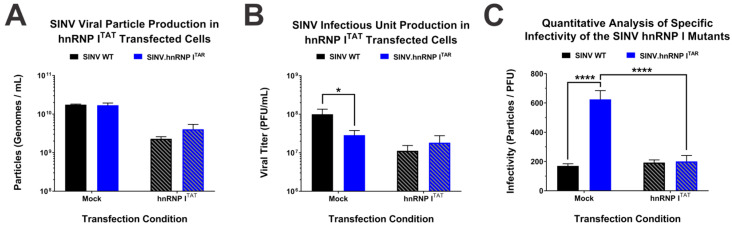
Reestablishment of the hnRNP–vRNA interaction restores viral particle infectivity. (**A**) Virus particles, as defined by the genome equivalents per ml, derived from either mock or hnRNP I^TAT^-transfected HEK293 cells were quantified via qRT-PCR. (**B**) Paired viral titer analysis of the samples examined in (A) as measured using standard plaque assays. (**C**) Quantitative analysis of virus-specific infectivity, as measured by the ratio of particles per infectious unit, for the samples described in the above panels. Quantitative data shown are the means of three independent infections, with the error bars representing the standard deviation of the means. Statistical significance as determined by Student’s t-test is indicated above the specific comparisons (with * ≤ 0.05; **** ≤ 0.001).

Precisely how the loss of the hnRNP I protein–RNA binding negatively impacts the SINV infection despite enhancing the structural protein expression has always been an interesting yet puzzling question. Since structural protein expression is directly linked to viral particle assembly, we sought to determine whether or not the production of viral particles was negatively impacted by the loss of hnRNP I binding [45,46]. To address this research question, we measured the total particle production via the detection of genome equivalents by way of qRT-PCR. Briefly, control-transfected and hnRNP I^TAT^-expressing cells were infected with either SINV.WT or SINV.hnRNP I^TAR^, and tissue culture supernatants were collected at 24 hpi. The number of viral genomic RNAs was then measured via standard curve qRT-PCR to determine the number of viral particles. As shown in Figure 6A, the loss of hnRNP I binding does not negatively affect the particle production, as there is no difference in particle numbers between SINV.WT and SINV.hnRNP I^TAR^ in either the presence or absence of hnRNP I^TAT^. Consistent with our above data the expression of hnRNP I reduced the particle production relative to the control-transfected cells, as there was an approximately half-log decrease in particle production for both SINV.WT and SINV.hnRNP I^TAR^. 

While the production of total viral particles was seemingly unaffected by the loss and restoration of hnRNP I binding, we hypothesized that the viral particle function, as defined by the capacity of a viral particle to complete the viral lifecycle, is negatively impacted by the loss of hnRNP I binding and subsequent structural protein overexpression. To define the functional potential of the viral particles generated in the presence and absence of the hnRNP I interaction, we measured the titer of the viral particles (Figure 6B) and determined the specific infectivity of the particles by calculating the ratio of particles-per-PFU for the individual samples. In this instance, a higher specific infectivity value means that it takes more particles to make a single plaque forming unit, meaning the viral particle population has poor infectious potential.

As shown in Figure 6C, SINV.WT particles derived from control-transfected cells exhibited an infectivity ratio of approximately 170:1 particles-per-PFU, whereas SINV.hnRNP I^TAR^ was significantly less infectious, with a particle-per-PFU ratio of greater than 600:1. Nonetheless, when the hnRNP I protein–RNA interaction was restored via the BIV-TAR/TAT system, the specific infectivity of SINV.hnRNP I^TAR^ significantly improved to a ratio of 200:1 and exhibited an infectivity ratio highly similar to that of SINV.WT. It is notable that SINV.WT particles exhibited a similar infectivity ratio regardless of whether they were produced in control-transfected or hnRNP I^TAT^-transfected cells. 

Altogether these data indicate that the particle functionality, as measure by the infectious potential of the population, is negatively impacted by the direct loss of hnRNP I binding and not the loss of specific primary nucleotide sequences or secondary structures in the SINV 3′UTR. Moreover, these data infer that while the particle production and viral titer may be generally reduced in systems that express high levels of hnRNP I, the infectious potentials of wild-type viral particles are unperturbed. 

### 3.6. The Loss of hnRNP I Binding Does Not Negatively Impact Particle Assembly or Structure

**Figure 7:**
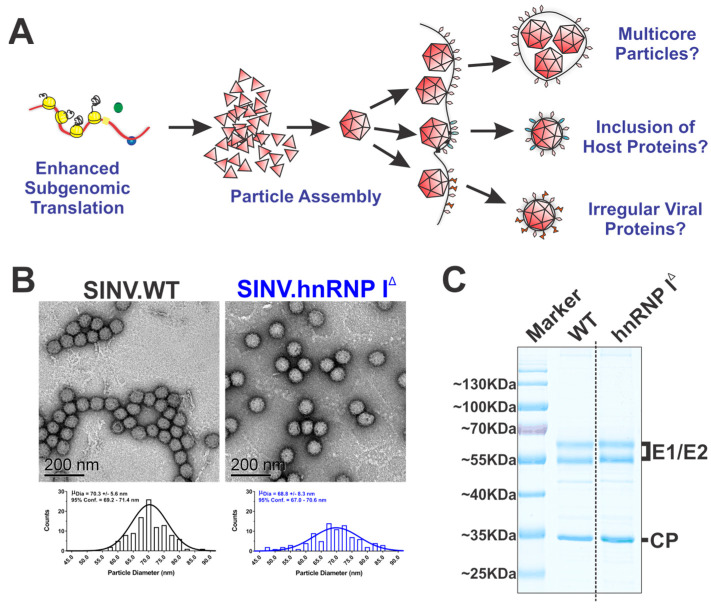
Loss of hnRNP I binding does not negatively impact the viral morphology or composition. (**A**) A graphic model of several working hypotheses as to how increased structural protein expression leads to poor particle infectivity. (**B**) Representative TEM micrographs of wild-type or SINV.hnRNP I^TAR^ particles purified via low-speed low-temperature centrifugation. Below each micrograph is a histogram of measured particle diameters with the mean and 95% confidence intervals reported inset to each graph. (**C**) Concentrated SINV.WT and hnRNP I interaction site deletion mutant viral particles were resolved via SDS-PAGE gel and stained with Coomassie blue. Data shown are representative of multiple independent viral preps. (**C**) The dashed line is indicative of where the gel was cropped and merged to remove intervening lanes for the final presented image.

As reported above, the loss of hnRNP I binding negatively impacted the specific infectivity of the viral particles. In light of these data, we hypothesized that the overexpression of SINV structural proteins leads to the formation of viral particles with decreased infectious potential, either through the formation of aberrant multicore viral particles, the inclusion or exclusion of host or viral proteins, or the production of irregular viral proteins during infection (as diagrammed in Figure 7A). To test this hypothesis, we set about characterizing the viral particles produced by wild-type SINV and SINV.hnRNP I^TAR^ in the presence and absence of hnRNP I^TAT^. 

The production of multicore particles would readily explain our previous observations, in that a single PFU would be composed of multiple genome equivalents, as several nucleocapsid cores would be packed into an envelope, resulting in a poor specific infectivity, as measured by the particle-per-PFU ratio [47]. To this end, we examined the morphologies of wild-type and hnRNP I interaction-deficient viral particles via transmission electron microscopy (TEM). As shown in Figure 7B, the overall morphologies of viral particles derived from hnRNP I binding and nonbinding SINVs were highly similar, and multicore particles were not observed. Curiously, the quantitative analysis of the particle diameter indicates that viral particles derived in the absence of hnRNP I binding exhibited increased heterogeneity, albeit to a minor extent. 

As the formation of multicore particles was not observed in the absence of hnRNP I binding, we next characterized the protein composition of the viral particles. Briefly, low-speed purified viral particles were denatured and analyzed via SDS-PAGE and the total protein content was visualized by Coomassie staining. As shown in Figure 7C, the viral particles produced in the presence and absence of hnRNP I binding were highly similar, and no significant unexpected proteins were observed. The quantitative analysis of the ratios of the viral glycoproteins to capsid protein provides further evidence against the formation of multicore particles, as the ratios between the particle populations are highly consistent. 

Notwithstanding the products of these efforts being largely negative data in regard to our hypothesis, these data were informative, as they effectively rule out the possibility that gross particle defects were arising due to increased structural protein expression. Nonetheless, from these data we cannot rule out that the malformation or misprocessing of the viral structural proteins during assembly negatively impacts the viral particle function.

### 3.7. The Loss of hnRNP I Binding Negatively Impacts the Early Stages of the Viral Lifecycle

Although the viral particles derived from the hnRNP I binding-deficient mutant are less infectious, the mechanism behind why they are poorly infectious is yet to be known. During the viral lifecycle there are several points with high potential to influence the specific infectious potential of a viral particle, and importantly these alphaviral lifecycle events can be parsed apart at certain points to determine where in the lifecycle the particles are functioning poorly. As our data above strongly indicates that the viral replication and gene expression are not explicitly negatively impacted by the loss of hnRNP I binding, it can be reasonably concluded that these events are not the primary defects leading to poor infectivity. As such, we hypothesized that an earlier event in the viral lifecycle was responsible for the observed deficits in specific infectivity. 

**Figure 8:**
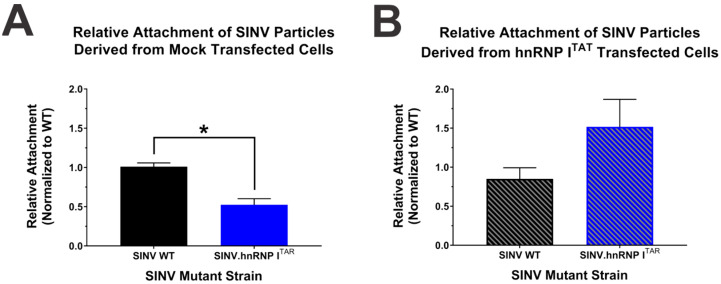
Loss of hnRNP I binding negatively impacts viral particle attachment. Quantitative analysis of viral attachment via qRT-PCR of total RNAs extracted from HEK293 cells that were incubated with viral particles derived from either (**A**) mock-transfected or (**B**) hnRNP I^TAT^-transfected HEK293 cells. Quantitative data shown are the means of three independent attachment assays, with the error bars representing the standard deviation of the means. Statistical significance as determined by Student’s t-test is indicated above the specific comparisons (with * ≤ 0.05).

To test our hypothesis, we quantitatively examined the first step of the viral lifecycle, which is the viral attachment to the cell. To accomplish this, we exposed HEK293 cells to either SINV.WT or SINV.hnRNP I^TAR^ particles derived from control-transfected or hnRNP I^TAT^-transfected cells at 4 °C for one hour to allow for attachment without entry or internalization of the viral particles. Paired tissue culture monolayers were then processed in parallel to generate input and bound samples, with the bound samples being generated from exposed monolayers that were extensively washed to remove unbound particles prior to RNA extraction. The viral RNAs from the input and bound samples virus were quantitatively assessed by qRT-PCR to determine the relative binding of the viral particles via the retention of genome equivalents. As shown by Figure 8A, the particles derived from infections lacking the hnRNP I interaction bound approximately two-fold less to cells relative to the particles derived from SINV.WT infection. Nonetheless, SINV.hnRNP I^TAR^ particles derived from hnRNP I ^TAT^-transfected cells bound equivalently to SINV.WT (Figure 8B). 

These data confirm our hypothesis that the loss of hnRNP I binding negatively impacts an early event in the viral lifecycle, resulting in poor specific infectivity. This assertion is evidenced by the reestablished particle attachment, which correlates with the above restoration of the infectivity of SINV.hnRNP I^TAR^ in hnRNP I^TAT^-transfected cells.

### 3.8. Deglycosylation of the hnRNP I Mutant Particles Does Not Impact Their Infectivity

The alphaviral entry pathway is initiated and governed by the viral glycoproteins through their engagement with the host receptor during attachment [47,48,49]. As our data are indicative of a defect at the level of the cell attachment, we hypothesized that the viral glycoproteins may be somehow altered in the absence of hnRNP I binding due to the overexpression of structural proteins during late infection. The viral glycoproteins are known to be post-translationally modified during their maturation process, including being palmitoylated and glycosylated as they traffic to the cell membrane for later envelopment of the nascent nucleocapsid cores [50,51,52,53,54]. As glycosylation has been previously identified as a major contributor to cell attachment, we prioritized efforts to examine the impact of glycosylation on the hnRNP I mutant particle function. 

**Figure 9:**
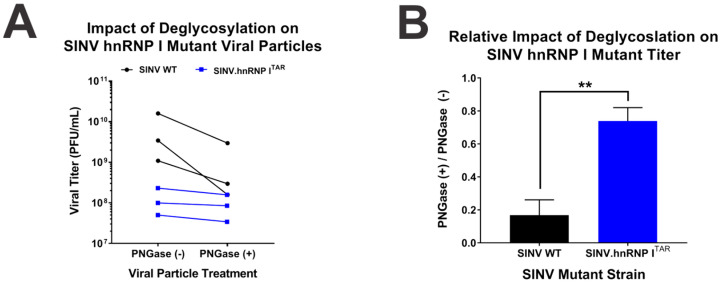
The loss of hnRNP I binding negatively impacts the glycosylation of the viral glycoproteins. (**A**) SINV.WT or SINV.hnRNP I^TAR^ viruses were incubated in the presence or absence of PNGase F overnight at room temperature under nondenaturing conditions. After treatment the viral titer was quantified and the change in viral titer is presented for each pairwise sample. (**B**) The relative impact of deglycosylation, as determined by the average ratio of treated and untreated samples. Quantitative data shown are the means of three independent PNGase F assays, with the error bars representing the standard deviation of the means. Statistical significance as determined by Student’s t-test is indicated above the specific comparisons (with ** ≤ 0.01).

To define the extent to which glycosylation differences were contributing to the observed deficits in particle function, we enzymatically deglycosylated SINV.WT and SINV.hnRNP I^TAR^ viral particles and assessed their infectious potentials. Concisely, aliquots of the viral particles were either mock-treated or treated with PNGase F under native protein conditions overnight, and the viral titer was subsequently assessed. The deglycosylation of SINV.WT particles via PNGase F negatively impacted the viral titer, as evidenced by a decrease of approximately 5-fold (as depicted in Figure 9A). In contrast, there was little to no decrease in the apparent viral titer when SINV.hnRNP I^TAR^ viral particles were treated with PNGaseF. Indeed, comparing the relative effects of the deglycosylation on SINV.WT and SINV.hnRNP I^TAR^ titer revealed that the deglycosylation did not appreciably affect the specific infectivity of the SINV.hnRNP I^TAR^ particles (Figure 9B). 

On the whole, these data strongly suggest that the differences in glycosylation may be responsible for the underlying defects observed following the loss of hnRNP I protein binding. Nonetheless, whether this is due to the absence of glycosylation or the presence of faulty glycosylation is unknown at this time. 

## 4. Discussion

As has been previously published, several host hnRNP proteins are known to interact with the SINV vRNAs during infection, with the hnRNP K, I, and M proteins interacting with discrete sites of the SINV subgenomic RNA [34]. The ubiquity and specificity of the hnRNP protein interactions was indicative of an important role during the SINV lifecycle. Nonetheless, due to the involvement of the hnRNP proteins in the synthesis and maturation of many cellular transcripts, RNAi- or CRISPR-based approaches would undoubtedly perturb the underlying cell system through the loss of hnRNP function. As shown here and published previously by our lab, an approach that enables the assessment of the contributions of the hnRNP proteins in the absence of an altered host system is to target the hnRNP–vRNA interaction sequence without disrupting the coding capacity of the virus. The application of this approach diminished the hnRNP–vRNA interactions, leading to significantly decreased viral growth kinetics in tissue culture models of infection. Curiously, the primary molecular defect associated with the disruption of the hnRNP interactions was increased structural protein expression, which positively correlated with decreased viral growth; however, the precise underlying mechanisms behind these phenomena were unknown. Altogether these observations raised several key questions, including the following: (1) Are the observed phenotypes due to the loss of hnRNP binding or due to disrupting the native RNA sequences? (2) How does the enhanced structural protein expression in effect result in a decreased viral titer?

To address our research questions, we utilized a modified protein tethering approach to reconstitute the hnRNP interaction in the absence of the native sequence. Since the current protein tethering methodologies are largely incompatible with use in coding regions, we focused our efforts on assessing the hnRNP I–vRNA interaction, as the primary interaction site for hnRNP I is located in the 3′UTR of the subgenomic RNA [34]. Specifically, the BIV-TAR element was incorporated into the vRNA at the site of the hnRNP I interaction site, where the BIV-TAR element could act as a highly specific binding site for proteins such as hnRNP I, provided the protein is tagged with a TAT peptide [35]. To confirm the capacity of the BIV-TAR/TAT system to reconstitute the hnRNP I–vRNA interaction, we assessed the interaction via quantitative co-immunoprecipitation. In the presence of hnRNP I^TAT^, the co-immunoprecipitation of the SINV.hnRNP I^TAR^ vRNA was equivalent to that of the wild-type interaction in the absence of hnRNP I^TAT^ and greater than that of the wild-type SINV in the presence of hnRNP I^TAT^. In other words, more SINV vRNA was pulled down during the forced interaction between SINV.hnRNP I^TAR^ and hnRNP I^TAT^ than that of SINV.WT in the presence of hnRNP I^TAT^. The underlying cause of the reduced wild-type SINV co-immunoprecipitation is unclear, and potentially due to several mechanisms. First, this could be due to the interaction between the BIV-TAR RNA and TAT fusion peptide being a stronger interaction than the native hnRNP I and vRNA interaction, resulting in greater occupancy and increased co-immunoprecipitation. In addition, the overexpression of hnRNP I could interfere with the immunoprecipitation by reducing the amount of RNA–protein complex binding relative to the total hnRNP I immunoprecipitation via an effective antibody dilution effect. 

Regardless, this system allowed us to directly compare the phenotypes observed between SINV infections with native hnRNP I interactions, those lacking native hnRNP I infections, and those with a forced hnRNP I interaction. As such, it is unsurprising that after confirming the validity of the BIV-TAR/TAT approach, we then tested the effect of the hnRNP I tethering on the viral growth kinetics. As observed before, there was a decrease in SINV.hnRNP I^TAR^ titer compared to SINV.WT in mock-transfected cells. However, this difference in infectious titer between wild-type and SINV.hnRNP I^TAR^ was not observed in hnRNP I^TAT^-transfected cells, indicating that the tethering of hnRNP I was capable of restoring the wild-type growth kinetics. 

Despite alleviating the growth defect resulting from the loss of the native hnRNP I interaction site, the overall titers for both the wild-type and interaction-deficient mutants were decreased in the presence of hnRNP I^TAT^ relative to mock-transfected cells. This was despite an experimental design that included using the same MOIs to infect either condition. Thus, the hnRNP I overexpression appears to be deleterious to SINV infection in a generalized manner. This observation is echoed by our assessments of viral gene expression, vRNA synthesis and accumulation, and viral particle production. As alluded to above, the steady-state levels of the hnRNP proteins, including hnRNP I, are likely important to the homeostasis of the host cell, and altering the levels of hnRNP I upwards or downwards may negatively impact the cytosolic environment. In support of this notion is the general observation that hnRNP I^TAT^-transfected cells looked morphologically abnormal and less confluent when compared with cells that had been mock-transfected. Accordingly, our leading hypothesis as to why viral titers were reduced overall is that hnRNP I overexpression negatively impacts host cell processes. In any case, the generalized impact of hnRNP I overexpression may be negated by ensuring that phenotypic comparisons are made with those between the viruses in a single transfection condition and not those between transfection conditions. 

### 4.1. Binding of hnRNP I Is Specifically Important for the Regulation of Viral Structural Protein Expression

Previously, we reported that disrupting the hnRNP I–vRNA interaction site led to increased structural protein expression; however, this prior effort examined viral gene expression in a limited manner late during infection, meaning the full picture of the potential role of hnRNP I in the regulation of viral gene expression throughout the lifecycle remained unknown. To enhance the understanding of the role of hnRNP I in the regulation of viral translation, we examined the viral gene expression with respect to time in systems with native hnRNP I interactions, those lacking native hnRNP I infections, and those with a forced hnRNP I interaction. 

In mock-transfected cells, there were no biologically significant differences in viral structural protein expression at 4, 8, or 12 hpi. However, at 16 hpi the SINV mutant lacking the hnRNP I interaction again exhibited enhanced structural protein expression. The timing of this effect may be indicative of the unavailability of the hnRNP I protein to the vRNAs, as hnRNP I relocalization to the cytoplasm has not likely occurred at these earlier stages of infection. As observed above for the viral growth kinetics, there was no difference in structural protein expression between SINV.WT and SINV.hnRNP I^TAR^ in hnRNP I^TAT^-transfected cells, despite a generalized decrease in viral structural protein expression. Hence, we conclude that the hnRNP I protein binding to the viral RNA is important for the regulation of the viral structural protein expression at late stages of viral infection.

### 4.2. Binding of hnRNP I Is Dispensable to SINV vRNA Synthesis and Accumulation

As the hnRNPs are RNA binding proteins that are involved in the processing of many cellular RNAs, it was imperative to examine the potential impacts of hnRNP I in viral RNA synthesis [29,30,55,56,57,58]. Previous studies have shown that knockdown or silencing of hnRNPs will cause decreases in alphaviral RNA synthesis; however, as discussed previously, this could be the result of disrupting the host cell biology through the loss of hnRNP function [12]. As with the viral gene expression, our prior efforts examining the role of the hnRNP proteins were limited to a singular time post-infection. Here, we expanded these analyses by examining the impact of hnRNP I on SINV replication and RNA synthesis with respect to time by using our model infection systems. Consistent with our prior examination of hnRNP I interaction-deficient mutants, we observed no explanative differences in RNA synthesis or accumulation for any of the vRNA species at any time post-infection in any of the conditions assessed. Collectively, these data infer that under conditions of equal infectious units, the viral RNA synthesis is unperturbed by the loss of hnRNP I binding.

Nonetheless, whether specific differences in viral RNA synthesis are present at the very early stages of viral infection remains unknown. As a primary difference between the native particles and those produced in the absence of hnRNP I binding is decreased infectious potential, one could envision a scenario where the viral RNA levels at the earliest stages of infection differ to a significant extent. The inequality of the viral particle function would, a priori, suggest that the viral RNA levels at the earliest instances of infection should differ by 2- to 3-fold, as per the observed differences in attachment and specific infectivity. However, these differences are not reflected by our data. There are several reasons as to why these differences are not propagated to the times post-infection assessed in this study, which were chosen on the basis of them representing times post-infection where all viral RNA species are readily detectable via qRT-PCR. First, it is unclear as to whether the infectious particles would effectively deliver their RNA cargos to the host system, thereby contributing them to the pool of cytoplasmic viral RNAs from which replication may proceed. To control for this possibility, the experimental designs were standardized to utilize equal numbers of infectious units (as PFU) to create a level playing field between the hnRNP I mutant and wild-type SINVs. Secondly, the alphaviral RNA synthesis kinetics are inherently very robust, and as such it remains possible that the RNA synthesis is capable of overcoming any early deficits through the inherent momentum of replication. 

Overall, from our data we conclude that the loss of hnRNP I binding does not significantly impact the RNA synthesis over time. However, as with our other data, there is an observable general impact of the hnRNP I overexpression on the vRNA synthesis. 

### 4.3. Loss of hnRNP I Binding Results in the Production of Poorly Infectious Virus Particles

All together, we can conclude that the phenotypic differences observed following the mutation of the hnRNP I interaction site were due directly to the loss of hnRNP I binding and not due to a loss of secondary RNA structures or primary sequences. Nonetheless, while our first major research question had been addressed, the question of how precisely an increase in structural proteins negatively effects viral infection remained elusive. To address this ongoing research question, we comparatively examined viral particles produced in the presence and absence of hnRNP I binding via the BIV-TAR/TAT system. 

A quantitative analysis of the viral particle production yielded an unexpected result—the increased viral structural protein expression did not correlate with a parallel increase in particle production. This observation was puzzling because despite there being more structural proteins to make more viral particles, there was no difference in particle production. Nonetheless, the differences in viral titer led to the hypothesis that the particles made in the absence of hnRNP I binding were less functional than wild-type viral particles. The virus-specific infectivity, as defined by the number of viral particles to infectious units, is a ready means by which the functionality of the viral particles in total may be assessed. These data presented above indicate that the viral particles produced in the absence of hnRNP I binding are poorly functional relative to the wild-type particles. In short, when there was no hnRNP I–vRNA interaction, many more particles were needed to make one infectious unit, and when the hnRNP I interaction was restored through the BIV-TAR/TAT system, the number of viral particles per infectious unit was similar to that of the wild-type SINV. Not only do these data reinforce the conclusion that the direct loss of hnRNP I binding is the specific cause of the observed mutant phenotype, these data provide valuable insight towards the elucidation of the underlying mechanism as to why there are decreased viral growth kinetics. 

We have established so far that hnRNP I is important to the regulation of the viral structural protein expression, and without that hnRNP I–vRNA interaction, there is an influx of structural proteins at the later stages of infection relative to the wild-type infection. Since alphaviral infections rely heavily on host processes to develop mature virions, these excess structural proteins could overwhelm the host biology and create a bottleneck in virus production. This in turn could create poorly functioning viral particles via several different mechanisms, which we alluded to in detail in the Section 3 Results. Notably, many of our efforts were designed to identify whether these overt defects were revealed wild-type-like phenotypes for the particles produced in the absence of the hnRNP I interaction. Nonetheless, in this case even the negative data were meaningful data, as they narrowed down the potential causes of the defective particles. 

Despite being able to rule out the formation of multicore particles or malformed particles, there was still no clear explanation of why these particles were poorly infectious. The molecular data presented here indicate that an early event of the viral lifecycle is negatively impacted by the loss of hnRNP I binding. By turning to the beginning of the alphaviral lifecycle and examining the viral attachment, we determined that the viral particles produced in the absence of hnRNP I binding were less able to bind to the host cells in the tissue culture models of infection. Importantly, this reduced attachment is ‘fixed’ when hnRNP I binding is restored via the BIV-TAR/TAT system. The reduced attachment to the host cell strongly suggested that the viral glycoproteins of the mutant particles were stoichiometrically inferior, malfunctioning, or malformed. The examination of the viral particle composition did not reveal altered capsid-to-glycoprotein ratios, indicating that the mutant viral particles were likely not lacking viral glycoproteins on the whole. As the alphaviral glycoproteins mature, they are post-translationally processed prior to their incorporation into the viral particles [50,51,52]. Of these potential post-translational modifications, the glycosylation of the E1 and E2 glycoproteins has been previously established to directly influence the viral attachment to the host cell, and the alphavirus glycosylation site mutants are poorly infectious owing largely to the altered host cell attachment [59]. Importantly, the deglycosylation of SINV viral particles generated in the presence and absence of hnRNP I binding reveals the difference in the glycosylation states to be a primary difference between the two particle populations. As the SINV particles derived from wild-type infection were sensitive to deglycosylation, whereas those generated in the absence of hnRNP I binding were insensitive, the defective particles formed during enhanced structural protein expression may lack or possess erroneous glycosylation profiles. Further studies are ongoing to determine precisely how an increase in structural protein expression results in this phenotype, whether the phenotype is the result of a bottleneck during processing or an active host response to infection, and whether this phenotypic defect is caused by altered glycosylation or a lack thereof.

It is worth noting that in addition to the defects in particle function related to glycosylation, other defects may also be present and may contribute to the phenotype observed during the loss of hnRNP I binding. These include aspects of infectivity related to viral lifecycle events prior to and after host cell attachment. For instance, our research has previously established that encapsidated host factors and viral RNA features, such as the 5′ cap structure, influence the particle infectivity [60,61]. Whether or not these phenomena are also altered in response to the increased structural protein expression is unknown at this time. 

### 4.4. Is hnRNP–vRNA Binding a Host Response to Infectio, or the Recruitment of a Pro-Viral Factor?

The sum of our observations raises an interesting question—is the repression of the viral translation via hnRNP I binding beneficial or detrimental to the virus? On face value, the molecular impacts of the hnRNP I binding, in that the viral structural protein expression is reduced, are reminiscent of an anti-viral response by the host. However, the output of the increased structural protein expression is the formation of poorly functioning viral particles, which infers that the hnRNP I interaction is beneficial to the viral infection through a complex means that maintains the functional integrity. The engagement of other host RNA-binding proteins to the alphaviral RNAs has been established to be largely pro-viral. Thus, we posit that the engagement of the hnRNP proteins to the viral RNAs is pro-viral in nature due to the body of knowledge regarding alphaviral RNA-binding protein interactions, as well as the summative phenotype resulting from the loss of hnRNP I binding presented here. However, further work is needed to fully understand the precise roles of cellular RNA-binding proteins during viral infection, including the likely reality that the consequences of the RNA-binding protein function is redefined during infection through post-translational modifications or the formation of contextually novel ribonucleoprotein complexes on viral RNAs. 

## Data Availability

Not applicable.

## Supplementary Materials

The following supporting information can be downloaded at: https://www.mdpi.com/article/10.3390/v14071423/s1, Figure S1: hnRNP ITAT Expression Alters Cell Morphology and Negatively Impacts Cell Division.
