# Supplementary material for: Binding of hnRNP I–vRNA Regulates Sindbis Virus Structural Protein Expression to Promote Particle Infectivity
Source: Viruses. 2022 Jun 28;14(7):1423. doi: 10.3390/v14071423 (PMC9318202; doi:10.3390/v14071423)
Supplement: Supplementary file 1 [file viruses-14-01423-s001.zip › viruses-1717003-supplementary.pdf]

SUPPLEMENTAL FIGURE S1

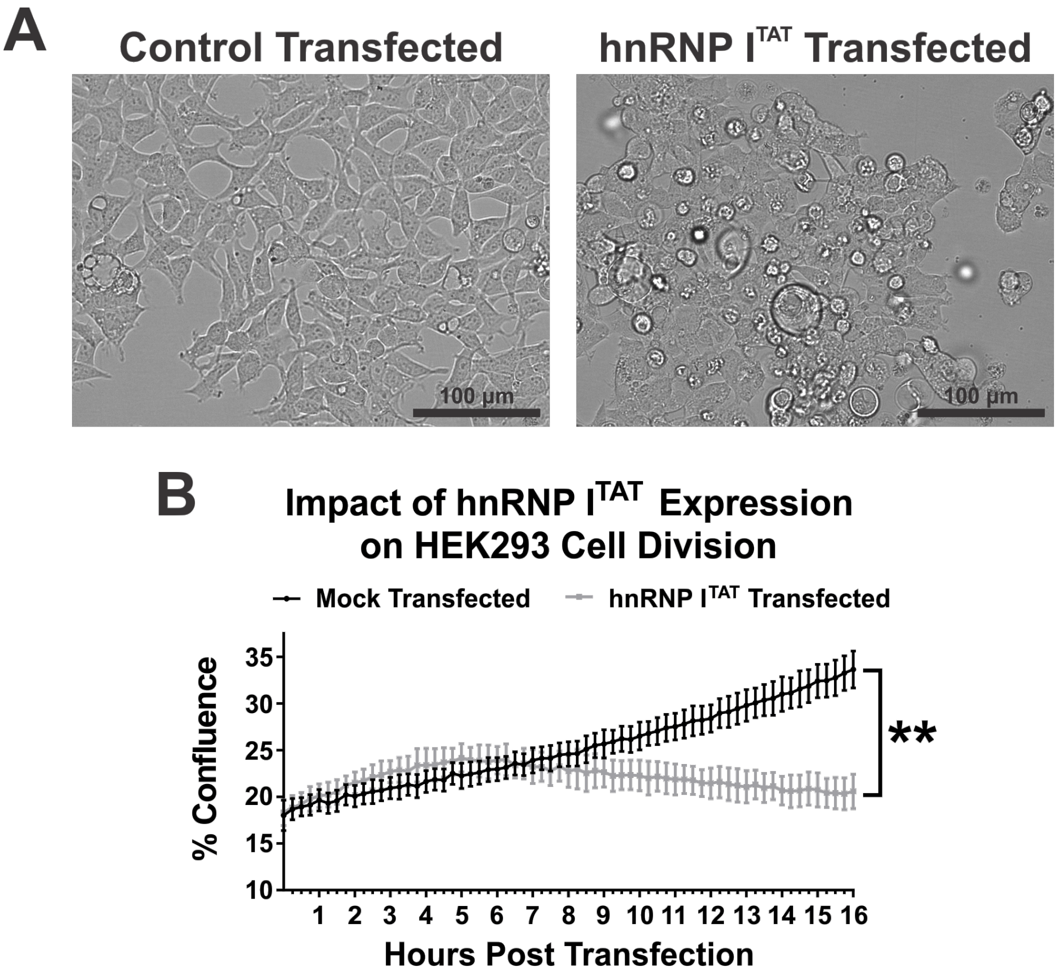

Supplemental Figure S1- hnRNP I^{TAT} Expression Alters Cell Morphology and Negatively Impacts Cell Division. (A) Representative bright field micrographs of control transfected and hnRNP I^{TAT} expression plasmid transfected HEK293 cells. Micrographs were taken using a Cytation 1 imaging multi-mode reader using a 20X objective at 16 hours post transfection. Micrographs were taken using identical imaging settings, and scale bars may be found inset on each micrograph. (B) Cell division, as per % confluence determined via digital phase contrast analysis using the Cytation 1 imaging multi-mode reader, was quantitatively assessed with respect to time. Quantitative data shown is the means of at least 8 biological replicates, with the error bards representing the 95% confidence intervals of the data points. Statistical significance, as determined by Area Under the Curve analysis is indicated alongside the specific comparisons (with ** ≤ 0.01).
